# Ozone Exposure Induces Pulmonary Microbiota Dysbiosis and Associated Inflammatory Responses in Mice

**DOI:** 10.3390/toxics14080736

**Published:** 2026-08-21

**Authors:** Ya Wang, Laibao Zhuo, Yue Du, Yuxuan Chai, Jiayin Li, Keyang Han, Juan Li, Weidong Wu

**Affiliations:** 1 School of Public Health, Henan Medical University, Xinxiang 453003, China; wangya2021@zjc.edu.cn (Y.W.); zhuolb@hotmail.com (L.Z.); duyue3711@163.com (Y.D.); 15738831310@163.com (Y.C.); 17839869389@163.com (J.L.); hankeyang2022@126.com (K.H.); 171019@xxmu.edu.cn (J.L.); 2 School of Health Nursing, Zhenjiang College, Zhenjiang 212028, China

**Keywords:** ozone, lung injury, pulmonary microbiota, inflammatory factors

## Abstract

Ozone (O_3_) is a prevalent environmental pollutant that can induce oxidative stress and respiratory epithelial injury. Dysregulation of pulmonary microbiota homeostasis plays an important role in the progression of lung damage and inflammatory responses. However, there remains a lack of systematic research on the effects of O_3_ exposure on pulmonary microecology and its potential association with pulmonary inflammation. In this study, twenty-three SPF-grade C57BL/6N male mice (aged 6–7 weeks) were randomly divided into a filtered air control group (*n* = 11) and an O_3_ exposure group (*n* = 12). Mice in the O_3_ group underwent whole-body inhalation exposure to 1 ppm O_3_ for 4 h daily over 8 consecutive weeks, while control mice were maintained under identical conditions with filtered air exposure. The 1 ppm concentration (equivalent to 0.2–0.3 ppm in humans) and 8-week daily 4 h exposure regimen was selected to model the 2-month summer high-O_3_ season with typical afternoon O_3_ peaks. The results showed that the O_3_-exposed group exhibited marked inflammatory infiltrates in the lung, as well as significantly elevated macrophage inflammatory protein-2 (MIP-2) and tumor necrosis factor-alpha (TNF-*α*) in bronchoalveolar lavage fluids compared with controls. Pulmonary microbiota sequencing revealed that O_3_ exposure significantly elevated the Shannon index of pulmonary microbiota in mice, and four differential genera including *Deinococcus*, *Aerococcus*, *Enterococcus* and *Proteiniphilum* were identified. Correlation analysis revealed that MIP-2 was significantly correlated with *Aerococcus*. In conclusion, the findings of this study suggest that exposure to O_3_ induces inflammation and significant changes in the microbial composition of the lungs, which provides an experimental basis for further investigating mechanisms linking microbiota dysbiosis to lung injury triggered by ambient air pollutants.

## 1. Introduction

Ozone (O_3_) is a major atmospheric pollutant of global concern, primarily formed from photochemical reactions involving nitrogen oxides and volatile organic compounds in the atmosphere [1]. In recent years, ground-level O_3_ concentrations have continued to rise due to the combined effects of climate change and human activities, including industrial emissions and vehicular exhaust. In China, O_3_ accounted for 47.9% of air pollution exceedance days in 339 cities, with an annual average concentration of 145 μg/m^3^, well above the WHO recommended daily maximum 8 h mean threshold of 100 μg/m^3^ for O_3_ [2]. Therefore, the detrimental outcomes of O_3_ exposure to human health have attracted widespread attention.

The respiratory system is particularly vulnerable to the effects of air pollution. The 2019 Global Burden of Disease (GBD) study indicates that long-term O_3_ exposure is associated with an increased risk of death from chronic obstructive pulmonary disease (COPD), accounting for more than 0.365 million premature deaths worldwide [3]. Short-term O_3_ exposure significantly impairs lung function in patients with asthma, with the damage being particularly pronounced in male patients, highlighting the sex-specific susceptibility to oxidative stress [4]. As a strong oxidative pollutant, O_3_ can rapidly penetrate the respiratory tract and trigger excessive reactive oxygen species (ROS) accumulation, which disrupts the dynamic balance of pulmonary oxidative stress and the antioxidant defense system [5]. Furthermore, exposure to 1 ppm O_3_ can induce airway hyperresponsiveness, alveolar inflammation, and tissue damage, thereby causing acute damage to the respiratory barrier, through the generation of ROS and activation of inflammatory signaling pathways [6]. Another study revealed that treatment with interleukin 33 (IL-33) neutralizing antibody markedly improved lung function, inflammatory cell infiltration, and immune response dysregulation induced by O_3_ exposure [7]. Therefore, exploring the underlying mechanisms of O_3_-induced lung injury is of significant practical importance.

Microbiota maintain normal physiological homeostasis and play critical roles in host immune regulation. Epidemiological evidence suggests that alterations in the pulmonary microbiota are associated with pulmonary function and lung immune homeostasis [8,9,10]. For instance, a cohort study has indicated that long-term particulate matter 2.5 (PM_2.5_) exposure alters the composition and structure of the lung microbiome, thereby affecting pulmonary function and increasing the susceptibility to respiratory infections [11]. Toxicological studies have further revealed that silica exposure triggers lung dysbiosis and activates the phosphatidylinositol 3-kinase (PI3K)/AKT pathway, ultimately resulting in pulmonary fibrosis [12]. Accumulating evidence has demonstrated that changes in the composition and abundance of pulmonary microbiota may occur long before clinical symptoms emerge, offering opportunities for early diagnosis and treatment, through non-invasive microbiome-based biomarkers [13]. These findings provide important clues to explore the mechanism underlying O_3_-induced lung injury.

Although the pulmonary toxicity of O_3_ has been increasingly recognized, the precise role of lung microbiota dysbiosis in O_3_-induced inflammatory injury remains largely unexplored. Therefore, we formulated the central hypothesis that chronic O_3_ exposure disrupts the composition and functional homeostasis of the pulmonary microbiota, leading to the enrichment of specific opportunistic pro-inflammatory taxa. We further hypothesized that this dysbiosis was associated with local innate immune activation and may participate in the aggravation of lung tissue injury. To test this hypothesis, this study was designed with three specific objectives: (1) to evaluate O_3_-induced pulmonary histopathological damage and quantify the levels of key pro-inflammatory cytokines including macrophage inflammatory protein-2 (MIP-2) and tumor necrosis factor-alpha (TNF-*α*) in bronchoalveolar lavage fluids; (2) to systematically characterize the changes in pulmonary microbial diversity, community structure, and predicted metabolic functions using 16S rRNA high-throughput sequencing; and (3) to identify differential microbial genera that were significantly correlated with the inflammatory responses via integrative correlation analysis. By achieving these objectives, we aimed to elucidate the potential link between pulmonary microbiota disturbance and O_3_-triggered lung inflammation, thereby providing novel microbial-based evidence and potential therapeutic targets for the prevention and intervention of air pollutant-induced respiratory diseases.

## 2. Materials and Methods

### 2.1. Laboratory Animals

Twenty-three specific-pathogen-free (SPF)-grade C57BL/6N male mice aged 6–7 weeks were purchased from Beijing Vital River Laboratory Animal Technology Co., Ltd. (Beijing, China). All mice were housed six per cage under standard laboratory conditions with a 12 h/12 h light–dark cycle, constant temperature of 23–26 °C, and relative humidity of 40–60%. Mice were maintained in cages with a maximum housing density of six mice per cage, resulting in 4 cages in total (3 cages with 6 mice and 1 cage with 5 mice) to accommodate all 23 animals. Autoclaved ultrapure water and standard chow were provided ad libitum. After three weeks of acclimatization, mice were randomly allocated to two experimental groups: the clean air control group (CON, *n* = 11) and the 1 ppm O_3_ exposure group (O_3_, *n* = 12). Cage assignments were balanced between the two groups to minimize potential cage-related confounding effects. Only male mice were included in this study, based on two primary considerations. First, as depicted above, epidemiological evidence has demonstrated that O_3_-induced lung function impairment is more pronounced in male patients, indicating greater susceptibility in males to O_3_-related oxidative stress and pulmonary inflammation, which was the primary mechanistic focus of this investigation. Second, the use of a single sex allowed us to minimize potential confounding effects of the estrous cycle on pulmonary immune and inflammatory responses, thereby reducing inter-individual variability and enhancing the reproducibility and statistical robustness of our findings. All animal experimental procedures and animal welfare interventions were strictly conducted in accordance with the institutional animal care and use guidelines. The entire animal study protocol was fully reviewed and formally approved by the Animal Ethics Committee of Henan Medical University (XYLL-2022-0174), which ensured that all experimental operations complied with the principles of animal welfare, reduction and standardized experimental animal management.

### 2.2. O_3_ Exposures

In this study, mice were exposed via a whole-body inhalation approach in a stainless-steel dynamic exposure system designed by our research group and calibrated by a professional company. This system comprises two identical, independently controlled chambers (an O_3_ exposure chamber and a HEPA-filtered control chamber), each with a volume of 300 L (internal dimensions: 800 mm × 600 mm × 625 mm), to simulate real-world environmental exposure scenarios. Up to four cages can be placed in each exposure chamber. In this system, O_3_ was generated by high-voltage corona discharge ionization of high-purity oxygen (>90%), and the chamber concentration was maintained at 1 ppm using a PID feedback control loop monitored by a Thermo Scientific Model 49i UV photometric O_3_ analyzer (Thermo Fisher Scientific, Franklin, MA, USA; detection limit: 1 ppb). The corona discharge method was selected for its ability to generate stable, high-concentration O_3_ with minimal nitrogen oxide byproducts, which was confirmed by the UV absorption analyzer’s selectivity for O_3_ at 253.7 nm. Temperature (23–26 °C) and relative humidity (40–60%) were maintained constant in both chambers. The system was calibrated before each exposure session.

Mice in the O_3_ group underwent whole-body inhalation exposure to 1 ppm O_3_ for 4 h daily over 8 consecutive weeks, while control mice were maintained under identical conditions with filtered air exposure. The 1 ppm concentration was selected based on allometric scaling, corresponding to a human-equivalent ambient level of approximately 0.2–0.3 ppm during summer peak pollution episodes [14]. This 8-week duration was deliberately chosen to simulate the prolonged seasonal summer high-O_3_ episode, which typically persists for approximately two months (e.g., June through July) in many regions with severe O_3_ pollution. The daily 4 h exposure was designed to coincide with the typical duration of photochemically driven O_3_ peaks.

To better mimic human outdoor activity patterns and avoid interference from volatile compounds released by feed and bedding volatile substances, food and bedding materials were completely removed from the exposure chamber during each O_3_ treatment session. After the completion of daily exposure, all mice were transferred back to the standard SPF animal rearing room, with sufficient and free access to sterile food and drinking water throughout the entire experimental cycle. In this present study, following the final O_3_ exposure, mice were deeply anesthetized by intraperitoneal injection of pentobarbital sodium (50 mg/kg body weight). After confirming the loss of the pedal withdrawal reflex, euthanasia was completed by cervical dislocation to ensure rapid and humane death.

### 2.3. Lung Tissue Collection and Bronchoalveolar Lavage

Lung tissue collection and bronchoalveolar lavage (BAL) were performed immediately after animal euthanasia. Briefly, mice were thoroughly disinfected with 75% ethanol under sterile conditions to avoid exogenous contamination. The right main bronchus was tightly ligated with sterile surgical thread and transected, and the isolated right lung tissues were immediately immersed and fixed in 4% paraformaldehyde solution for subsequent histopathological staining. For BAL following animal euthanasia, the left lung was subjected to BAL three times using 1000 μL of pre-chilled sterile phosphate-buffered saline (PBS) per lavage to fully harvest the BAL fluids (BALFs) in the left lung. After each slow infusion of PBS, the BALF was gently aspirated to prevent mechanical injury to lung tissue, and the recovered BALF from each lavage was collected into sterile centrifuge tubes. Approximately 80% of the total infused volume was recovered. The collected BALF samples were aliquoted and snap-frozen in liquid nitrogen and preserved at −80 °C in a freezer. Enzyme-linked immunosorbent assay (ELISA) and 16S rRNA gene sequencing were completed in single batches within 2 weeks after sample collection.

All 23 mice (CON: *n* = 11; O_3_ group: *n* = 12) underwent BAL and lung tissue harvest. Sample allocation for subsequent analyses was as follows: Pulmonary microbiota 16S rRNA gene sequencing was performed on samples from 11 mice (4 CON mice and 7 O_3_-exposed mice). For ELISA quantification of inflammatory cytokines (MIP-2 and TNF-*α*), 7 mice per group were randomly selected from the 23 animals, considering sufficient recovered BALF volume and statistical power. For histopathological assessment via H&E staining, 3 mice per group were randomly chosen as representative samples.

### 2.4. Histopathological Staining

Lung tissue samples were immediately fixed in 4% paraformaldehyde solution for 24 h at room temperature after sampling for hematoxylin–eosin (H&E) staining. After sufficient fixation, lung tissues underwent dehydration through a graded ethanol series, followed by transparent treatment with xylene, and were embedded in paraffin to prepare paraffin tissue blocks. The paraffin blocks were trimmed and serially sliced into continuous sections with a thickness of 5 μm using a rotary microtome. After dewaxing in xylene and gradual rehydration via descending concentrations of ethanol, the sections were stained with hematoxylin for 5 min and rinsed thoroughly with running tap water. Differentiation was performed using 1% hydrochloric acid alcohol for several seconds, followed by bluing treatment in tap water. Subsequently, the sections were counterstained with eosin working solution for 1–2 min. After staining, the sections were dehydrated once again with gradient ethanol, cleared with xylene, and finally sealed permanently with neutral balsam. Qualitative observation and image capture of histopathological morphological changes in lung tissues, including inflammatory cell infiltration, alveolar structural damage and tissue edema, were carried out under an optical light microscope.

### 2.5. ELISA

The levels of pro-inflammatory cytokines, including TNF-*α* and MIP-2, in the supernatants of mouse BALF were quantitatively detected based on the sandwich enzyme immunoassay technique using commercially available ELISA kits (Cusabio Co., Ltd., Wuhan, China) following the manufacturer’s instructions. Briefly, the microplate was pre-coated with a capture antibody specific to the target proteins, herein, TNF-*α* and MIP-2. After adding standards or samples, the antigen bonded to the immobilized antibody. Then, a biotin-conjugated detection antibody was added, followed by HRP-conjugated avidin, forming the “capture antibody–antigen–biotin detection antibody–HRP avidin” complex. Finally, 3,3′,5,5′-tetramethylbenzidine (TMB) substrate was added for color development, and the absorbance was read at 450 nm. The color intensity was directly proportional to the target concentration of tested proteins.

### 2.6. Microbiota Profiling

To characterize the composition and functional profiles of pulmonary microbiota in the BALF, 16S rRNA gene high-throughput sequencing was performed on the Illumina NovaSeq 6000 platform (Illumina Inc., San Diego, CA, USA). Raw sequencing reads were processed using the VSEARCH algorithm as implemented in the QIIME2 package for rigorous quality filtering, accurate paired-end read assembly, and effective chimera removal to eliminate invalid sequences and ensure data reliability. Taxonomic classification was subsequently carried out to assign representative sequences to corresponding microbial taxa. The qualified clean sequences were further clustered into operational taxonomic units (OTUs) based on a 97% sequence similarity threshold, and the final valid sequencing data were obtained for subsequent microbial diversity analysis. Alpha- and beta-diversity analyses were implemented to evaluate the richness, uniformity and overall structural variation in pulmonary microbial communities. Furthermore, functional prediction of the microbial community was conducted using the PICRUSt2 algorithm to annotate the potential biological metabolic functions of the differential microbiota and explore the underlying functional alterations related to O_3_ exposure.

### 2.7. Statistical Analysis

Differences in BALF inflammatory cytokine levels and alpha-diversity indices of pulmonary microbiota between the control and O_3_ exposure groups were assessed using the non-parametric Wilcoxon rank-sum test. Based on the normalized genus-level taxonomic abundance table, permutational multivariate analysis of variance (PERMANOVA) was performed using the weighted UniFrac distance matrix to quantitatively evaluate overall differences in microbial community structure and composition between groups. Differentially abundant bacterial taxa with significant intergroup differences were identified using linear discriminant analysis effect size (LEfSe) with default screening thresholds. Spearman’s rank correlation analysis was further conducted to systematically evaluate the potential associations between the relative abundances of differential microbial genera and BALF total protein or inflammatory cytokine levels. Functional differences in pulmonary microbiota between groups were analyzed using the Wilcoxon rank-sum test. A two-sided *p* value < 0.05 was considered statistically significant in all statistical tests. All statistical analyses were performed using SPSS version 22.0 (SPSS Inc., Chicago, IL, USA), and all experimental figures were generated using GraphPad Prism version 5.0 (GraphPad Software, San Diego, CA, USA).

## 3. Results

### 3.1. Morphological Features of the Lung After O_3_ Exposure

**Figure 1:**
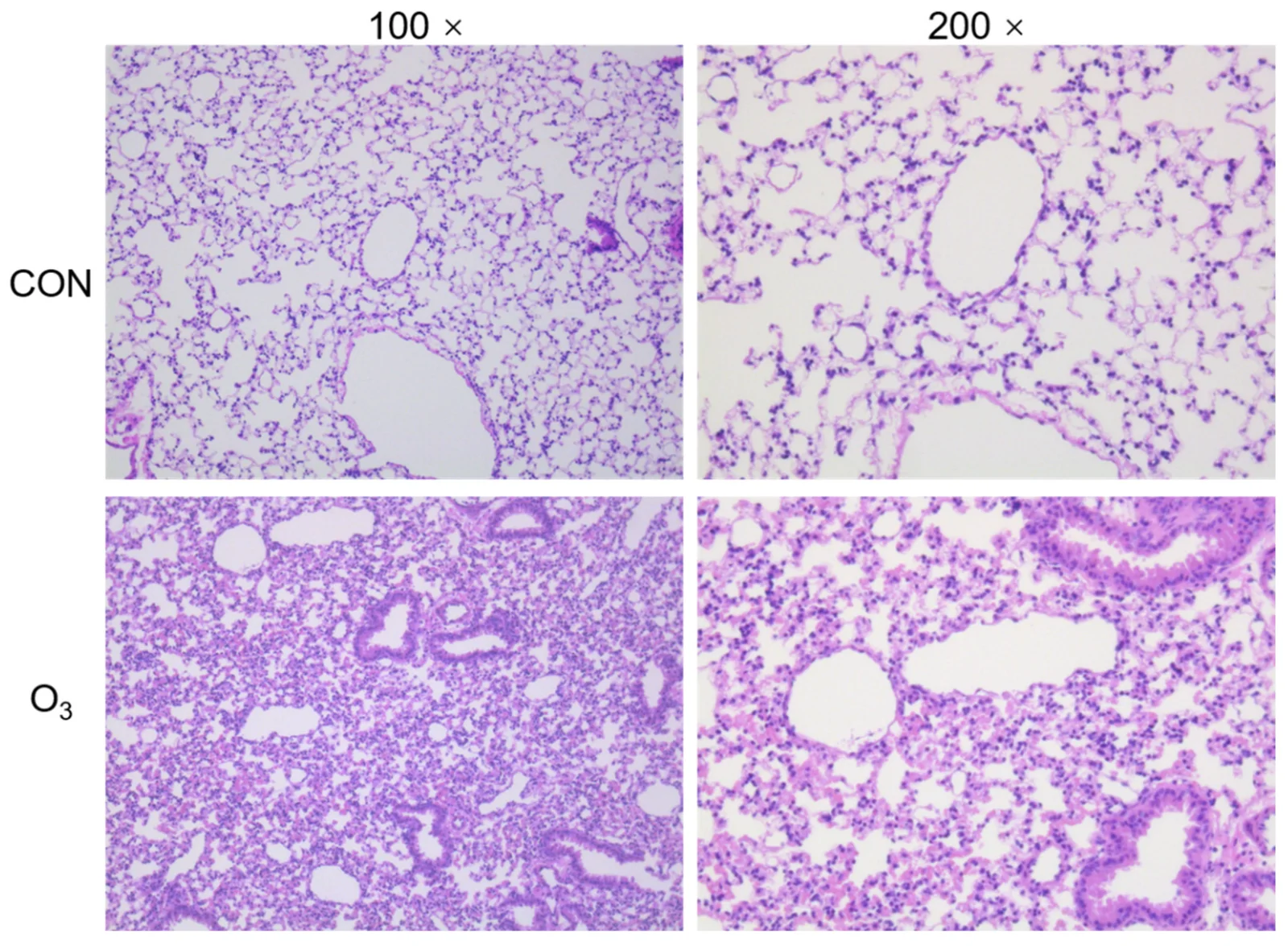
Morphological features of the lung after O_3_ exposure in mice. Representative H&E-stained lung sections from the filtered air control group (CON) and the O_3_ exposure group (O_3_). C57BL/6N male mice were exposed to 1 ppm O_3_ or filtered air for 4 h/day for 8 consecutive weeks (*n* = 3 mice per group for histology). Abbreviations: CON, control; O_3_, ozone; H&E, hematoxylin and eosin.

Pulmonary histopathological alterations induced by O_3_ were evaluated via H&E staining (Figure 1). Mice in the CON exhibited intact lung structure with uniform alveolar septa and negligible inflammatory infiltration. In contrast, mice exposed to 1 ppm O_3_ showed marked inflammatory cell infiltration in the lung interstitium, accompanied by interstitial thickening and hyperplasia. These histological alterations demonstrated that O_3_ exposure induced significant pulmonary inflammatory injury and structural disorder in mouse lungs.

### 3.2. BALF Inflammatory Cytokine Levels After O_3_ Exposure

**Figure 2:**
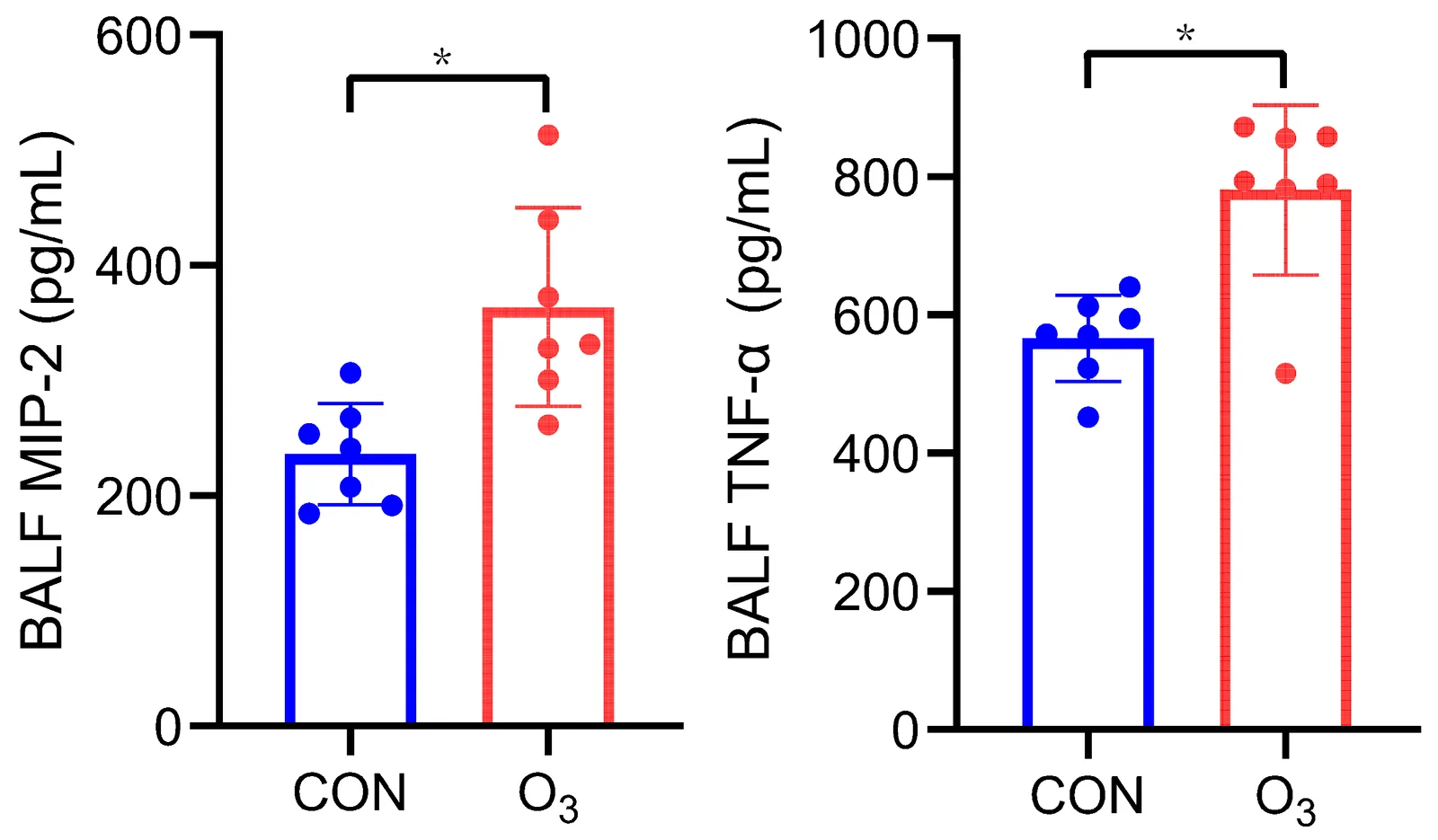
Inflammatory cytokine levels in bronchoalveolar lavage fluids (BALFs) after O_3_ exposure. Concentrations of macrophage inflammatory protein-2 (MIP-2) and tumor necrosis factor-alpha (TNF-*α*) in BALF from C57BL/6N male mice exposed to 1 ppm O_3_ or filtered air (CON) for 4 h/day for 8 weeks (*n* = 7 per group). Bars represent the mean, and error bars show SD. Statistical significance was determined using the Wilcoxon rank-sum test. * *p* < 0.05 versus the CON. Abbreviations: BALF, bronchoalveolar lavage fluid; CON, control; MIP-2, macrophage inflammatory protein-2; O_3_, ozone; SD, standard deviation; TNF-*α*, tumor necrosis factor-alpha.

To further explore pulmonary inflammatory responses induced by O_3_ exposure, the concentrations of pro-inflammatory mediators in the BALF were measured via ELISA. MIP-2, as a critical chemokine, facilitates the recruitment of inflammatory cells into lung tissue [15], and TNF-*α* serves as a key pro-inflammatory mediator that amplifies local inflammatory signaling [16]. Compared with the CON, mice in the 1 ppm O_3_ exposure group exhibited elevated levels of chemokine MIP-2 and pro-inflammatory cytokine TNF-*α* in the BALF, with statistically significant differences (*p* < 0.05, Figure 2). These results indicated that O_3_ exposure triggered pulmonary inflammation, consistent with the histopathological alterations revealed by H&E staining.

### 3.3. Effects of O_3_ Exposure on Pulmonary Microbiota Diversity and Composition

**Figure 3:**
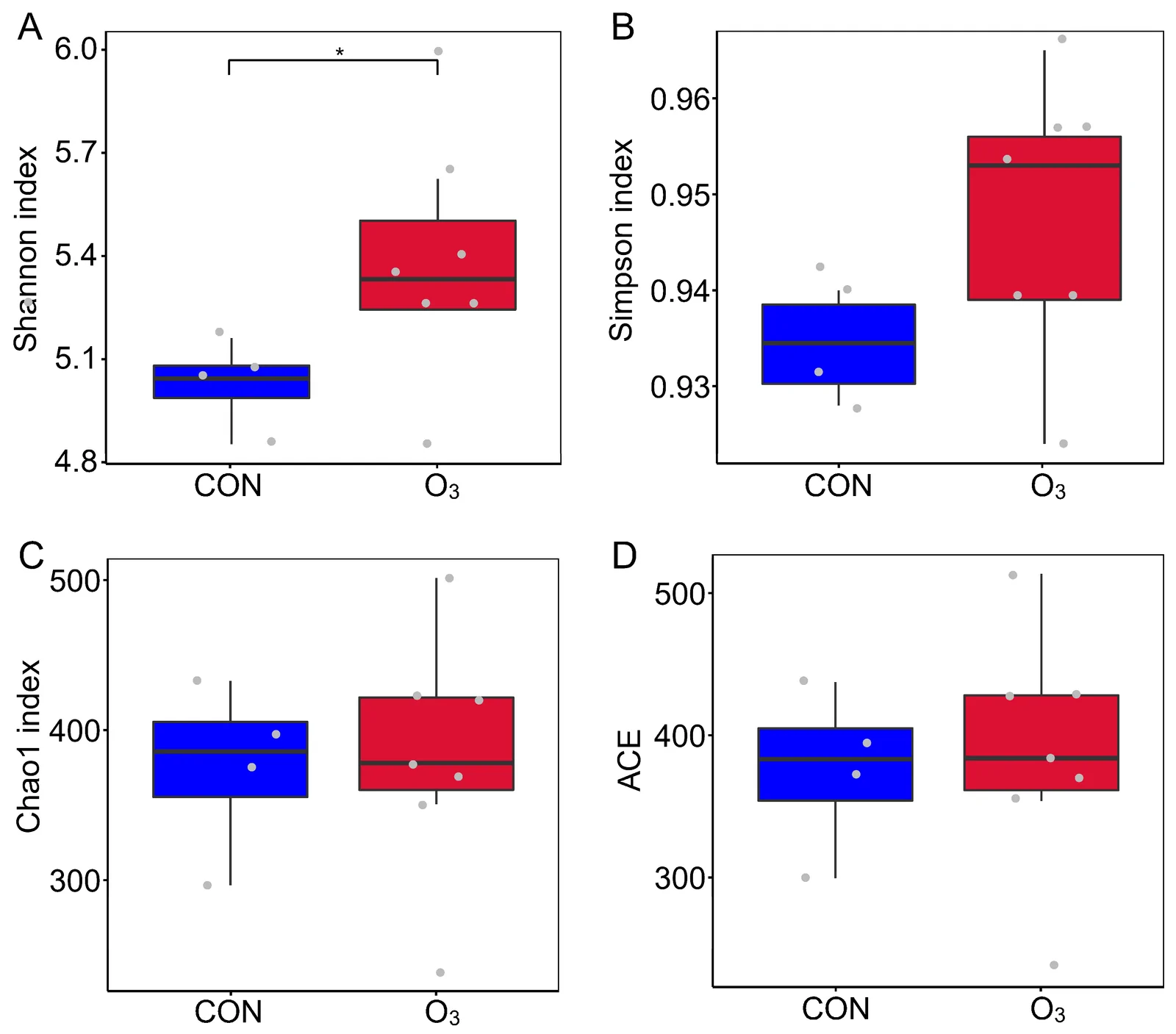
Alpha diversity of pulmonary microbiota after O_3_ exposure. Comparison of Shannon (**A**), Simpson (**B**), Chao1 (**C**) and ACE (**D**) indices between the filtered air control group (CON, *n* = 4) and the O_3_ exposure group (O_3_, *n* = 7). C57BL/6N male mice were exposed to 1 ppm O_3_ or filtered air for 4 h/day for 8 weeks. Box plots show the median (horizontal line) and interquartile range (box). Grey dots represent individual data points. Statistical significance was determined using the Wilcoxon rank-sum test. * *p* < 0.05 versus CON. Abbreviation: ACE, Abundance-based Coverage Estimator.

To comprehensively investigate the alterations in pulmonary microbial community structure and diversity induced by inhaled O_3_, high-throughput 16S rRNA gene sequencing was performed to systematically evaluate the overall diversity, richness and compositional characteristics of mouse pulmonary microbiota. The alpha-diversity analysis was applied to reflect the richness and homogeneity of individual microbial communities. As shown in Figure 3, the Shannon index was significantly increased in the O_3_ exposure group compared with the control group (*p* < 0.05), while the other alpha-diversity indices, including Simpson, Chao1, and ACE, showed no statistically significant differences between the two groups (*p* > 0.05).

**Figure 4:**
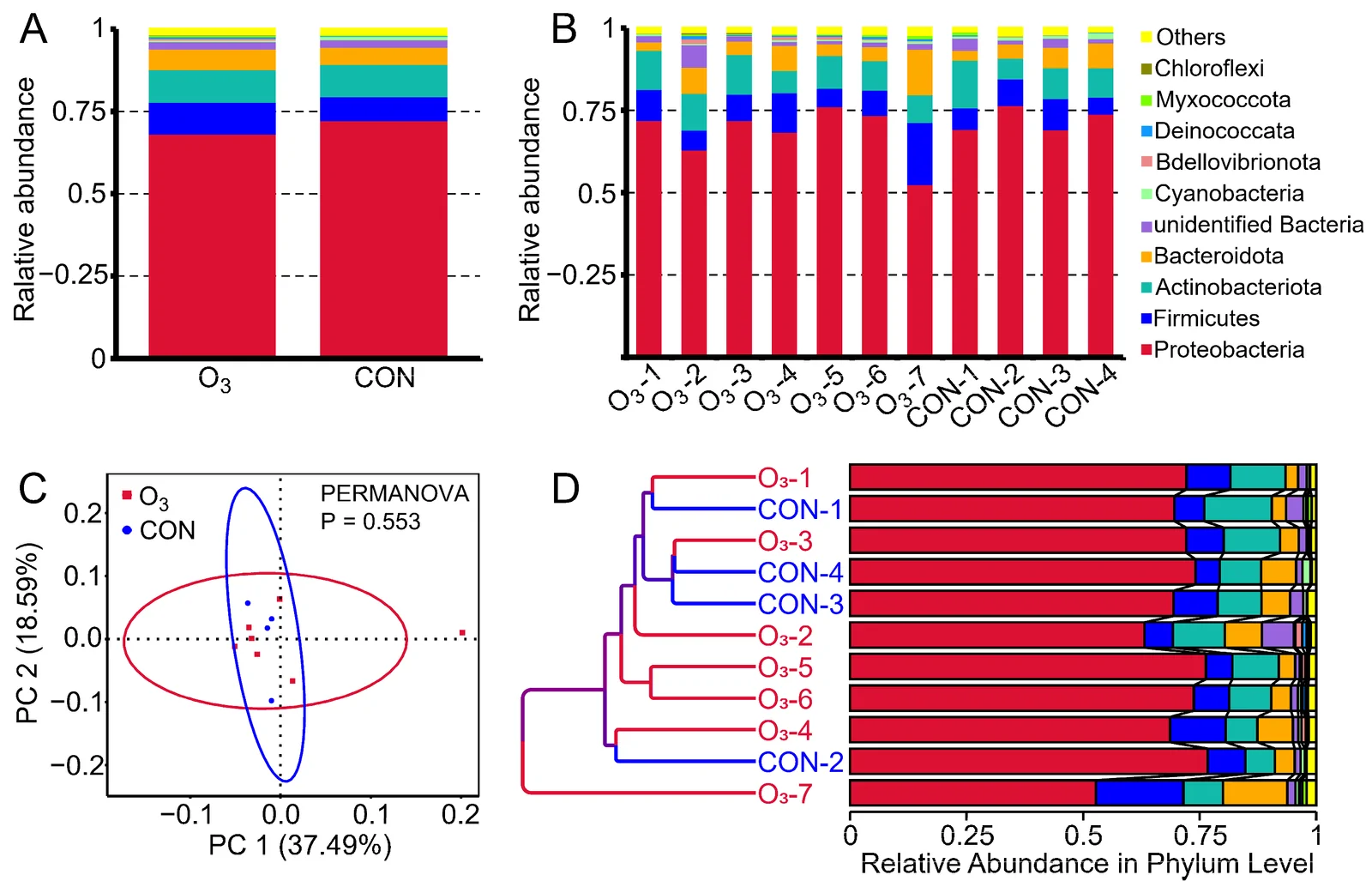
Composition and beta diversity of pulmonary microbiota after O_3_ exposure. (**A**,**B**) Stacked bar charts showing the relative abundance of bacterial phyla in each sample from the filtered air control group (CON, *n* = 4) and the O_3_ exposure group (O_3_, *n* = 7). (**C**) PCoA plot based on weighted UniFrac distances. Each point represents an individual mouse; red and blue 95% confidence ellipses correspond to the O_3_ group and CON group, respectively, to illustrate clustering. (**D**) Hierarchical clustering tree combined with species abundance distribution across different groups. C57BL/6N male mice were exposed to 1 ppm O_3_ or filtered air for 4 h/day for 8 weeks. Statistical differences in microbial community composition were evaluated using PERMANOVA based on Bray–Curtis distances with 999 permutations, which confirmed a significant difference between the two groups (*p* < 0.05). Abbreviations: CON, control; O_3_, ozone; PCoA, principal coordinate analysis; PERMANOVA, permutational multivariate analysis of variance.

Furthermore, microbial compositional analysis at the phylum level revealed subtle visual differences in the relative abundance of several dominant bacterial phyla between the CON and O_3_ exposure group (Figure 4A,B), although no statistical verification was performed for phylum-level differential analysis. Principal coordinate analysis (PCoA) based on weighted UniFrac distances of OTUs showed no obvious spatial separation of overall pulmonary microbial communities between the two groups (Figure 4C), and PERMANOVA analysis further confirmed no significant difference in the overall microbial community structure between CON and O_3_ group (*p* > 0.05). Notably, hierarchical clustering tree analysis (Figure 4D) presented a weak clustering trend for individual samples within the same group. For instance, CON1, CON3, and CON4 samples clustered on the upper branch of the tree. Collectively, these results indicated that chronic O_3_ exposure induced slight variations in the relative abundance of partial pulmonary bacterial phyla but failed to cause significant overall structural remodeling of pulmonary microbial communities.

### 3.4. Correlations Between Differential Genera of Pulmonary Microbiota and BALF Inflammatory Factors

**Figure 5:**
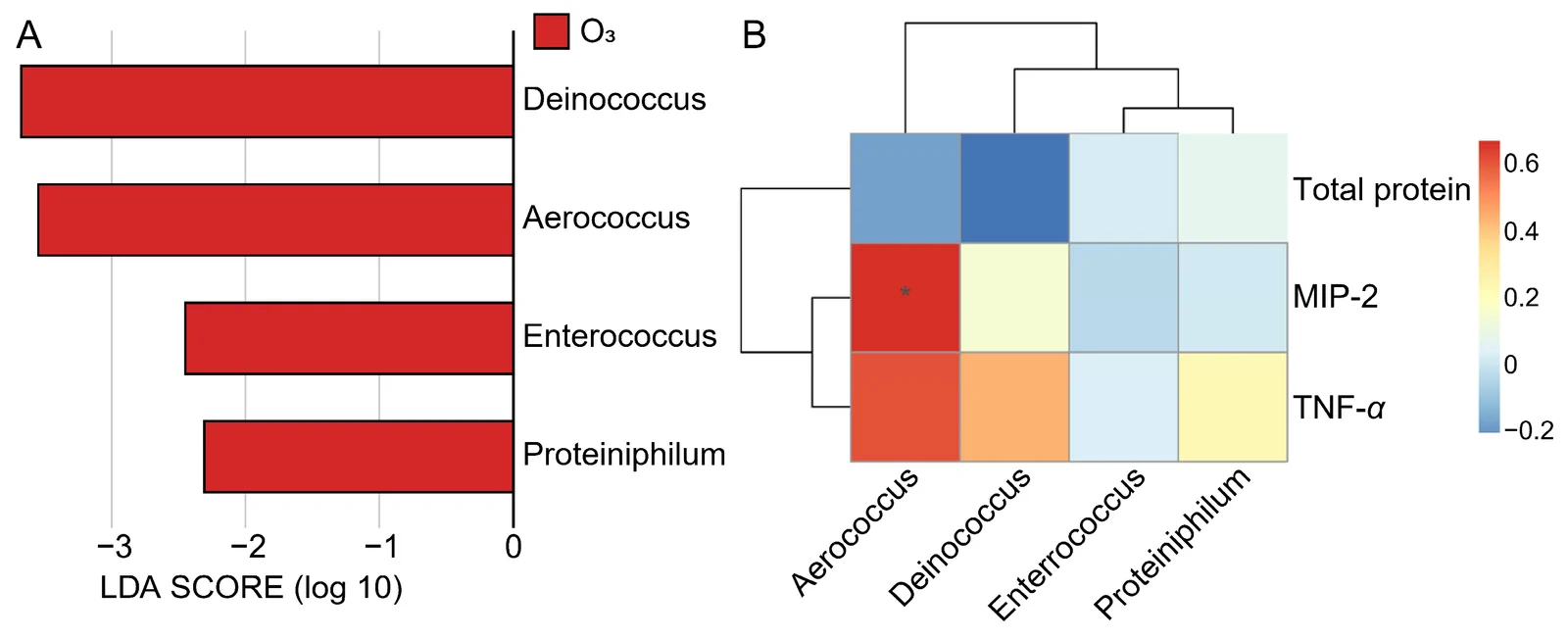
Correlation analysis between key differential microbial genera and inflammatory indicators after O_3_ exposure. C57BL/6N male mice were exposed to 1 ppm O_3_ or filtered air for 4 h/day for 8 weeks. (**A**) Linear discriminant analysis effect size (LEfSe) analysis showing microbial genera significantly enriched in the O_3_ group compared with the filtered air control group (CON), with an LDA score threshold >2. (**B**) Heatmap of Spearman’s rank correlation coefficients between the relative abundances of the four differential genera (*Deinococcus*, *Aerococcus*, *Enterococcus*, and *Proteiniphilum*) and BALF inflammatory markers (MIP-2 and TNF-*α*). * *p* < 0.05 indicates a significant correlation. Abbreviations: CON, control; O_3_, ozone; MIP-2, macrophage inflammatory protein-2; TNF-α, tumor necrosis factor-alpha; LDA, linear discriminant analysis; BALF, bronchoalveolar lavage fluid.

LEfSe analysis was applied to screen distinct microbial taxa and successfully identified four differential pulmonary genera between the control and O_3_ exposure groups, including *Deinococcus*, *Aerococcus*, *Enterococcus*, and *Proteiniphilum*, all of which were significantly enriched in the O_3_-exposed mice (Figure 5A). Spearman correlation analysis was further conducted to evaluate the potential relationships between these four differential genera and BALF inflammatory factors. As shown in Figure 5B, the abundance of *Aerococcus* displayed a significant positive correlation with the MIP-2 level (*p* < 0.05). No significant correlations were observed between the other three differential bacterial genera and the detected inflammatory markers in the BALF.

### 3.5. Effects of O_3_ Exposure on Functional Characteristics of Pulmonary Microbiota

**Figure 6:**
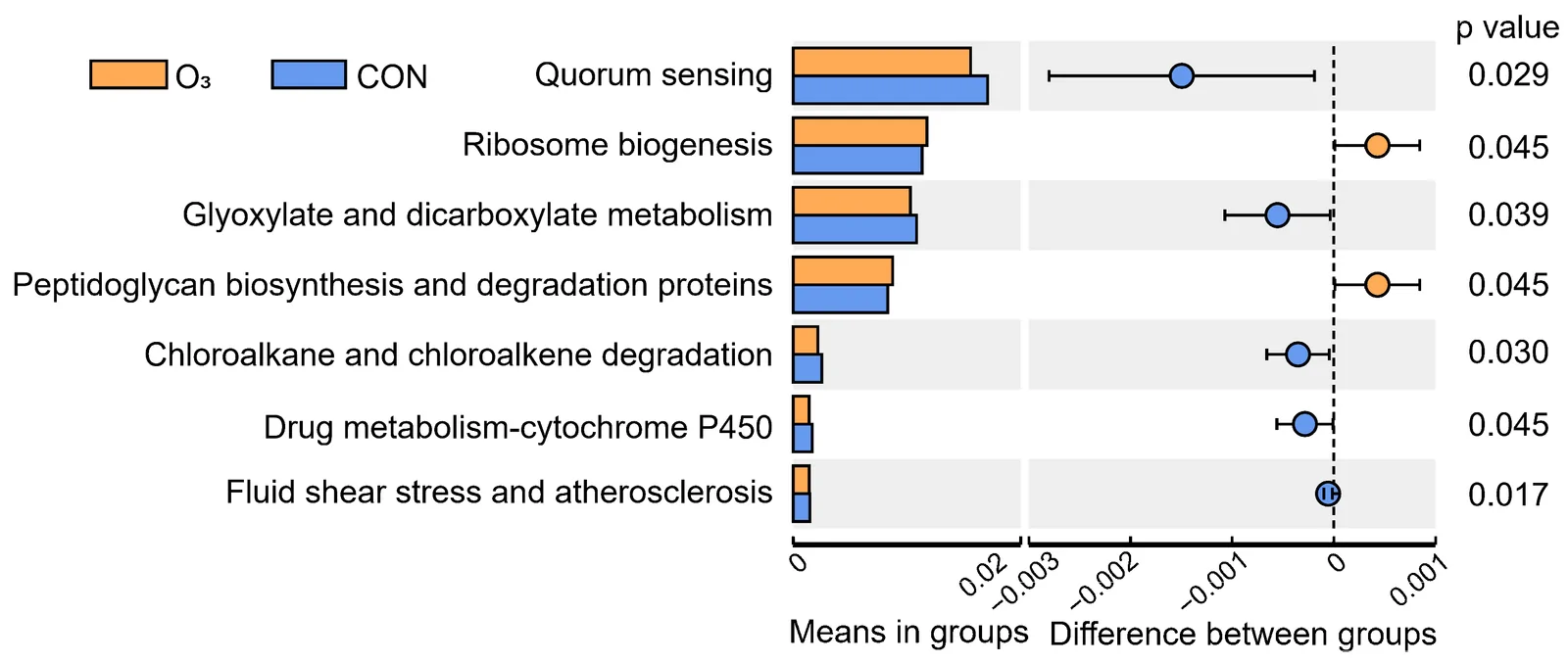
Effects of O_3_ exposure on functional characteristics of pulmonary microbiota in C57BL/6N mice. Male C57BL/6N mice were exposed to filtered clean air (control, CON) or 1 ppm O_3_ for 4 h per day for 8 consecutive weeks to evaluate changes in microbial functional profiles. Bar graphs display the relative abundance of differential Kyoto Encyclopedia of Genes and Genomes (KEGG) functional pathways predicted based on pulmonary microbial community composition between the two groups. All data are presented as mean ± standard deviation (SD). Wilcoxon rank-sum test was used for statistical analysis.

To further explore the potential functional alterations in lung microbiota induced by O_3_ exposure, we performed predictive functional enrichment analysis based on microbial community composition. A total of seven metabolic pathways exhibited significant differences between the control and O_3_-exposed groups (Figure 6). Notably, these differential pathways mainly involved suppressed bacterial quorum sensing, elevated ribosome biogenesis, and downregulated glyoxylate and dicarboxylate metabolism, suggesting that O_3_ exposure profoundly reshaped microbial metabolic profiles.

## 4. Discussion

We combined lung pathology, inflammation and microbiota data from experimental models to explore O_3_-induced lung injury and found that lung microbiota dysbiosis was closely associated with O_3_-triggered inflammation and injury. Our findings revealed a significant correlation between microbial compositional shifts (particularly the enrichment of *Aerococcus*) and the elevation of pro-inflammatory cytokines, suggesting that dysbiosis may be potentially associated with O_3_-associated pulmonary inflammatory responses.

O_3_ is a typical atmospheric irritant that readily penetrates the lower respiratory tract and causes cumulative oxidative toxic injury to the respiratory mucosa and delicate lung tissues. As a widespread urban air pollutant, ambient O_3_ imposes severe threats to public health and exacerbates respiratory dysfunction in both healthy and vulnerable populations [17,18]. Accumulating epidemiological evidence has consistently demonstrated that prolonged and high-concentration O_3_ exposure significantly impairs pulmonary function, elevates the incidence of multiple respiratory disorders, and increases hospitalization and mortality risks of pneumonia, COPD, asthma, and allergic rhinitis [13,19,20]. Long-term O_3_ pollution exposure has been widely recognized as a critical environmental risk factor driving the initiation and aggravation of various acute and chronic pulmonary diseases.

Toxicological evidence further indicates that O_3_ exposure at 1280 μg/m^3^ can induce obvious pulmonary pathological structural damage, mainly manifested as alveolar congestion, alveolar septum thickening, tissue edema and disordered pulmonary microstructure [2]. Such structural damage impairs the defensive function of the respiratory epithelium and renders the lung more susceptible to inflammatory stimulation. A body of animal experiments also supports that repeated O_3_ inhalation triggers progressive destruction of pulmonary tissue architecture [21,22]. In accordance with these previous findings, our histological observation confirmed that repeated O_3_ exposure could effectively induce apparent lung tissue injury and destroy the integrity of normal alveolar structure in mice, further verifying the direct pulmonary toxic effect of O_3_ exposure.

In addition to structural pathological damage, O_3_ exposure also provokes robust pulmonary inflammatory responses. Documented studies have revealed that O_3_ stimulation can effectively activate pulmonary innate immune responses and promote the massive recruitment and infiltration of inflammatory effector cells, primarily macrophages and neutrophils, in lung interstitial and alveolar spaces [23,24]. These activated immune cells further secrete and release abundant pro-inflammatory mediators, thereby significantly upregulating the expression levels of key inflammatory factors such as TNF-*α*, IL-6 and C-X-C motif chemokine ligand 1 (CXCL1) in the BALF [25]. In line with previous research results, the inflammatory detection results from this present study also confirmed the strong pro-inflammatory characteristic of O_3_ exposure. A large number of pro-inflammatory factors are subsequently released, which further aggravates local inflammatory damage and forms a sustained inflammatory microenvironment in the lung [26]. Therefore, we speculate that O_3_ exposure may lead to lung pathological changes by triggering local inflammatory responses.

The lung has long been regarded as a sterile organ. However, advances in high-throughput sequencing have confirmed the presence of a dynamic and complex microbial community in the lungs [27]. The homeostasis of lung microbiota is regulated by microbial immigration, inhalation, clearance and proliferation [28], and the composition and abundance of pulmonary microbial communities shift dynamically under different disease conditions, such as infection, chronic inflammation, and environmental toxicant exposure [29]. Disturbance of this delicate ecological balance can further disrupt respiratory immune homeostasis and aggravate local inflammatory responses. For instance, pathogenic *Proteobacteria*, especially *Haemophilus*, are increased in asthma and COPD patients, whereas Candida albicans accumulates in patients with cystic fibrosis (CF) [30,31,32]. Animal experiments also support the association between the lung microbiome and the progression of pneumonia. In BALB/c mice with *Fusarium graminearum* pulmonary infection, lung microbiota exhibited reduced *α*-diversity and dysbiosis, with increased *Staphylococcus* and decreased *Clostridium* [33]. Consistent with these findings, air pollutant exposure also disrupts pulmonary microbial homeostasis. A previous study in ICR mice found that PM_2.5_ exposure induced a 75.2% reduction in lung microbial diversity with elevated *Proteobacteria* and decreased *Bacteroidetes* [34]. Such air pollutant-triggered dysbiosis may act as an important intermediate link connecting environmental stimuli and pulmonary inflammatory injury. In support of these findings, our current study investigated lung microbiota changes following O_3_ exposure and confirmed that O_3_ exposure significantly altered the lung microbial profile in mice with four differentially abundant genera that may serve as potential biomarkers for O_3_-induced pulmonary injury, including *Deinococcus*, *Aerococcus*, *Enterococcus* and *Proteiniphilum*.

Lung microbiota dysbiosis alters local and systemic immune cell profiles and establishes a feedback circuit between immune cells and microbes, which in turn activates pulmonary immunity and induces abundant release of pro-inflammatory cytokines including chemokines and ROS [35]. Previous studies have indicated that environmental pollutant exposure can disrupt respiratory microbial homeostasis and further trigger pulmonary inflammatory responses [36,37,38]. For example, a study of 50 adult patients with community-acquired pneumonia (CAP) demonstrated that respiratory microplastic (MP) exposure was correlated with reduced respiratory microbiota *α*-diversity and elevated levels of inflammatory cytokines, including IL-2, IL-4, and IFN-*γ* [39]. Our study revealed a significant correlation between the pro-inflammatory chemokine MIP-2 and *Aerococcus*. *Aerococcus* is a genus of Gram-positive opportunistic bacteria commonly detected in environmental samples and respiratory tract microbiota. Previous evidence has demonstrated that increased abundance of *Aerococcus* can trigger robust inflammatory responses by elevating multiple pro-inflammatory mediators and promoting inflammatory cell infiltration [40,41]. In bovine and murine infection models, pathogenic *Aerococcus viridans* was capable of activating inflammatory signaling cascades and inducing marked secretion of TNF-*α*, IL-1*β* and IL-6 [42]. The expansion of this genus may amplify inflammatory signaling within the pulmonary microenvironment upon O_3_ stimulation. Such microbial disturbance provides a plausible mechanistic explanation for the sustained lung inflammation observed in exposed mice. O_3_-induced epithelial injury may create a favorable niche for the proliferation of opportunistic microbes such as *Aerococcus*. The interplay between impaired epithelial barrier and outgrowth of pro-inflammatory bacteria likely accelerates the progression of lung tissue damage. Given the established role of *Aerococcus* in driving inflammatory pathology, we hypothesize that O_3_-induced enrichment of *Aerococcus* may be associated with the progression of pulmonary inflammatory injury in mice, serving as a potential microbial link between air pollutant exposure and host immune activation. However, this correlative observation requires further functional validation.

The major strength of this research lies in revealing the correlation between O_3_ exposure-induced pulmonary microbiota disturbance and lung inflammation in a mouse model under controlled repeated exposure conditions. By combining 16S rRNA high-throughput sequencing of pulmonary microbiota and quantitative detection of BALF inflammatory factors, we identified *Aerococcus* as a key differential genus closely and positively associated with MIP-2. This finding offers a new microbial clue for understanding the pathogenic process of O_3_-mediated pulmonary injury and provides potential microbial targets for subsequent mechanistic research. Collectively, these findings expand the current understanding of the respiratory toxicological effects of ambient O_3_ exposure and preliminarily reveal a potential association between O_3_ pollution, alterations in pulmonary microbiota, and host pulmonary inflammatory responses.

Nevertheless, several limitations of the current work should be acknowledged. First, this study only employed male C57BL/6N mice. While this design minimized variability from sex-related hormonal fluctuations and focused on the more susceptible male phenotype, it limits the generalizability of our findings to female populations. Future studies should include both sexes to evaluate potential sex-specific differences in O_3_-induced pulmonary dysbiosis and inflammation. Second, this study used a single O_3_ concentration (1 ppm) and a single exposure duration (8 weeks). Although the 1 ppm dose was selected based on allometric scaling to approximate human-relevant exposure levels during summer pollution peaks, and the 8-week regimen was designed to model the prolonged seasonal high-O_3_ episode, a dose–response study with multiple concentrations and time points would provide a more comprehensive understanding of the relationship between O_3_ exposure, pulmonary microbiota alterations, and inflammatory responses. Such studies are warranted in future investigations. Third, while we have identified a significant correlation between *Aerococcus* enrichment and MIP-2 levels, we did not perform causal experiments (e.g., microbial transplantation, antibiotic depletion, or germ-free models) to confirm that the observed dysbiosis directly drives the inflammatory phenotype. The correlative nature of our findings precludes any definitive conclusions about causality. Fourth, we did not analyze microbial metabolites derived from lung microbiota, which limits our ability to explore downstream metabolic pathways through which differential microbes may affect pulmonary inflammation. Finally, all experimental data were obtained from mouse models. The results cannot be directly generalized to humans, and thus caution should be exercised when extrapolating these findings to humans, due to obvious interspecies differences in respiratory tract structure, microbiota colonization patterns and host immune responses. Despite these limitations, our study provides a solid foundation for future mechanistic and translational research. Future studies will integrate microbial metabolomics analysis for further in-depth mechanistic investigation.

## 5. Conclusions

In summary, this study demonstrates that chronic repeated O_3_ exposure induces significant pulmonary histopathological damage and a robust local inflammatory response, as evidenced by markedly elevated levels of the chemokine MIP-2 and the pro-inflammatory cytokine TNF-*α* in the BALF. Concurrently, 16S rRNA sequencing reveals that O_3_ exposure profoundly disrupts the pulmonary microbial ecosystem, characterized by decreased *α*-diversity, an altered overall community structure, and the significant enrichment of four differential genera—*Deinococcus*, *Aerococcus*, *Enterococcus*, and *Proteiniphilum*. Notably, Spearman correlation analysis identifies a significant positive association between the abundance of *Aerococcus* and MIP-2 levels, suggesting that this opportunistic genus may be associated with the amplification of inflammatory signaling within the pulmonary microenvironment. Furthermore, functional prediction indicates that O_3_ exposure perturbs key microbial metabolic pathways, including quorum sensing and glyoxylate/dicarboxylate metabolism, which may underlie the observed host–microbe interplay. Collectively, our findings provide novel integrative insights into the role of pulmonary microbiota dysbiosis in O_3_-triggered lung inflammation and lay a foundation for future mechanistic investigations targeting the inflammation–microbiota axis.

## Data Availability

The raw data supporting the conclusions of this article will be made available by the authors on request.

## Abbreviations

The following abbreviations are used in this manuscript:

O_3_	ozone
MIP-2	macrophage inflammatory protein-2
TNF-*α*	tumor necrosis factor-alpha
GBD	Global Burden of Disease
ROS	reactive oxygen species
IL-33	interleukin 33
PM_2.5_	particulate matter 2.5
PI3K	phosphatidylinositol 3-kinase
CON	control
BALF	bronchoalveolar lavage fluid
PBS	phosphate-buffered saline
HE	hematoxylin–eosin
ELISA	enzyme-linked immunosorbent assay
OD	optical density
OTUs	operational taxonomic units
PERMANOVA	permutational multivariate analysis of variance
LEfSe	linear discriminant analysis effect size
COPD	chronic obstructive pulmonary disease
CF	cystic fibrosis
CAP	community-acquired pneumonia
MP	microplastic
CXCL1	C-X-C motif chemokine ligand 1

## References

[1] Zhang J.J., Wei Y., Fang Z. (2019). Ozone Pollution: A Major Health Hazard Worldwide. Front. Immunol..

[2] Chen L., Jia Y., Su H., Chen L., Zhang P., Li D., Chen J. (2025). Sub-chronic exposure to ambient ozone induced liver and lung damage: Raveled by lung-liver axis. Front. Environ. Sci. Eng..

[3] Zhang Z., Wang C., Lin C., Wu Y., Wei J., Lu J., Chen B., Wu C., Zhang X., Yang Y. (2024). Association of long-term exposure to ozone with cardiovascular mortality and its metabolic mediators: Evidence from a nationwide, population-based, prospective cohort study. Lancet Reg. Health–West. Pac..

[4] Enweasor C., Flayer C.H., Haczku A. (2021). Ozone-Induced Oxidative Stress, Neutrophilic Airway Inflammation, and Glucocorticoid Resistance in Asthma. Front. Immunol..

[5] Wiegman C.H., Li F., Ryffel B., Togbe D., Chung K.F. (2020). Oxidative Stress in Ozone-Induced Chronic Lung Inflammation and Emphysema: A Facet of Chronic Obstructive Pulmonary Disease. Front. Immunol..

[6] Sokolowska M., Quesniaux V.F.J., Akdis C.A., Chung K.F., Ryffel B., Togbe D. (2019). Acute Respiratory Barrier Disruption by Ozone Exposure in Mice. Front. Immunol..

[7] Fang X., Pan B., Xie Y., Shao W., Li J., Han D., Hong X., Tu W., Zhao Y., Wu J. (2025). Mechanistic insights into ozone-induced asthma exacerbation: Role of oxidative stress and IL-33. J. Hazard. Mater..

[8] Zhou A., Lei Y., Tang L., Hu S., Yang M., Wu L., Yang S., Tang B. (2021). Gut Microbiota: The Emerging Link to Lung Homeostasis and Disease. J. Bacteriol..

[9] Li R., Li J., Zhou X. (2024). Lung microbiome: New insights into the pathogenesis of respiratory diseases. Signal Transduct. Target. Ther..

[10] Paudel K.R., Dharwal V., Patel V.K., Galvao I., Wadhwa R., Malyla V., Shen S.S., Budden K.F., Hansbro N.G., Vaughan A. (2020). Role of Lung Microbiome in Innate Immune Response Associated with Chronic Lung Diseases. Front. Med..

[11] Wang L., Cheng H., Wang D., Zhao B., Zhang J., Cheng L., Yao P., Di Narzo A., Shen Y., Yu J. (2019). Airway microbiome is associated with respiratory functions and responses to ambient particulate matter exposure. Ecotoxicol. Environ. Saf..

[12] Huo C., Jia Q., Jiao X., Jiang Q., Zeng X., Zhang J., Wang Y., Zhu Z., Tian L. (2025). Pulmonary microbiota affects silica-induced pulmonary fibrosis through activation of the PI3K/AKT-mediated senescence in alveolar epithelial cells. J. Hazard. Mater..

[13] King A. (2024). Exploring the lung microbiome’s role in disease. Nature.

[14] Nair A.B., Jacob S. (2016). A simple practice guide for dose conversion between animals and human. J. Basic Clin. Pharm..

[15] Qin C.C., Liu Y.N., Hu Y., Yang Y., Chen Z. (2017). Macrophage inflammatory protein-2 as mediator of inflammation in acute liver injury. World J. Gastroenterol..

[16] Webster J.D., Vucic D. (2020). The Balance of TNF Mediated Pathways Regulates Inflammatory Cell Death Signaling in Healthy and Diseased Tissues. Front. Cell Dev. Biol..

[17] Kim S.Y., Kim E., Kim W.J. (2020). Health Effects of Ozone on Respiratory Diseases. Tuberc. Respir. Dis..

[18] Deng Y., Wang J., Sun L., Wang Y., Chen J., Zhao Z., Wang T., Xiang Y., Wang Y., Chen J. (2023). Effects of Ambient O_3_ on Respiratory Mortality, Especially the Combined Effects of PM_2.5_ and O_3_. Toxics.

[19] Dearborn L.C., Hazlehurst M.F., Sherris A.R., Szpiro A.A., Day D.B., Loftus C.T., Blanco M.N., Adgent M.A., Andrade-Torres A.R., Ni Y. (2025). Early-Life Ozone Exposure and Asthma and Wheeze in Children. JAMA Netw. Open.

[20] Tang H., Du S., Tan Y., Su W., Niu Z., Sun J., Shao W., Fang X., Tang Z., Zhang D. (2026). The role of the nasal microbiome in linking ozone pollution to allergic rhinitis in children. J. Hazard. Mater..

[21] Russo R.C., Togbe D., Couillin I., Segueni N., Han L., Quesniaux V.F.J., Stoeger T., Ryffel B. (2025). Ozone-induced lung injury and inflammation: Pathways and therapeutic targets for pulmonary diseases caused by air pollutants. Environ. Int..

[22] Lee J.M., Meshanni J.A., Vayas K.N., Sunil V.R., Radbel J., Laskin J.D., Laskin D.L., Gow A.J. (2025). Inhaled ozone induces distinct alterations in pulmonary function in models of acute and episodic exposure in female mice. Toxicol. Sci..

[23] Guttenberg M.A., Vose A.T., Birukova A., Lewars K., Cumming R.I., Albright M.C., Mark J.I., Salazar C.J., Swaminathan S., Yu Z. (2023). Tissue-resident alveolar macrophages reduce O_3_-induced inflammation via MerTK mediated efferocytosis. bioRxiv.

[24] Oakes J.L., O’Connor B.P., Warg L.A., Burton R., Hock A., Loader J., Laflamme D., Jing J., Hui L., Schwartz D.A. (2013). Ozone enhances pulmonary innate immune response to a Toll-like receptor-2 agonist. Am. J. Respir. Cell Mol. Biol..

[25] Hsieh M.H., Chen P.C., Hsu H.Y., Liu J.C., Ho Y.S., Lin Y.J., Kuo C.W., Kuo W.S., Kao H.F., Wang S.D. (2023). Surfactant protein D inhibits lipid-laden foamy macrophages and lung inflammation in chronic obstructive pulmonary disease. Cell. Mol. Immunol..

[26] Chavda V.P., Bezbaruah R., Ahmed N., Alom S., Bhattacharjee B., Nalla L.V., Rynjah D., Gadanec L.K., Apostolopoulos V. (2025). Proinflammatory Cytokines in Chronic Respiratory Diseases and Their Management. Cells.

[27] Dickson R.P., Erb-Downward J.R., Martinez F.J., Huffnagle G.B. (2016). The Microbiome and the Respiratory Tract. Annu. Rev. Physiol..

[28] O’Dwyer D.N., Dickson R.P., Moore B.B. (2016). The Lung Microbiome, Immunity, and the Pathogenesis of Chronic Lung Disease. J. Immunol..

[29] Whiteside S.A., McGinniss J.E., Collman R.G. (2021). The lung microbiome: Progress and promise. J. Clin. Investig..

[30] Wang Z., Yang Y., Yan Z., Liu H., Chen B., Liang Z., Wang F., Miller B.E., Tal-Singer R., Yi X. (2020). Multi-omic meta-analysis identifies functional signatures of airway microbiome in chronic obstructive pulmonary disease. ISME J..

[31] Wang Z., Locantore N., Haldar K., Ramsheh M.Y., Beech A.S., Ma W., Brown J.R., Tal-Singer R., Barer M.R., Bafadhel M. (2021). Inflammatory Endotype-associated Airway Microbiome in Chronic Obstructive Pulmonary Disease Clinical Stability and Exacerbations: A Multicohort Longitudinal Analysis. Am. J. Respir. Crit. Care Med..

[32] Einarsson G.G., Comer D.M., McIlreavey L., Parkhill J., Ennis M., Tunney M.M., Elborn J.S. (2016). Community dynamics and the lower airway microbiota in stable chronic obstructive pulmonary disease, smokers and healthy non-smokers. Thorax.

[33] Ji J., Ye Y., Sun J., Sheng L., Li J., Yang J., Wu B., Wang Y., Gao X., Luo L. (2025). Homeostasis of Gut Microbiota Protects against Susceptibility to Fungal Pneumonia. Adv. Sci..

[34] Wang S., Zhou Q., Tian Y., Hu X. (2022). The Lung Microbiota Affects Pulmonary Inflammation and Oxidative Stress Induced by PM_2.5_ Exposure. Environ. Sci. Technol..

[35] Yang D., Xing Y., Song X., Qian Y. (2020). The impact of lung microbiota dysbiosis on inflammation. Immunology.

[36] Zheng Y., Zhang L., Tian J., Li N., Li Q., Li F., Meng J., Zhang Z., Yun X., Duan S. (2026). Lung microbiota dysbiosis mediates PM_2.5_-induced pulmonary inflammation through antibiotic-reversible mechanisms. J. Immunotoxicol..

[37] Xue Y., Chu J., Li Y., Kong X. (2020). The influence of air pollution on respiratory microbiome: A link to respiratory disease. Toxicol. Lett..

[38] Adar S.D., Huffnagle G.B., Curtis J.L. (2016). The respiratory microbiome: An underappreciated player in the human response to inhaled pollutants?. Ann. Epidemiol..

[39] Liu S., Zheng J., Lan W., Yang Z., Li M., Li J., Yu J., Yang S., Du J., Dong R. (2025). Microplastics exposed by respiratory tract and exacerbation of community-acquired pneumonia. The potential influences of respiratory microbiota and inflammatory factors. Environ. Int..

[40] Gilbert N.M., Choi B., Du J., Collins C., Lewis A.L., Putonti C., Wolfe A.J. (2021). A mouse model displays host and bacterial strain differences in Aerococcus urinae urinary tract infection. Biol. Open.

[41] Sahu K.K., Lal A., Mishra A.K., Abraham G.M. (2021). Aerococcus-Related Infections and their Significance: A 9-Year Retrospective Study. J. Microsc. Ultrastruct..

[42] Meng S., Xu M., Deng X., Zhu Z., Barkema H.W., Kastelic J.P., Cobo E.R., Han B., Liu G. (2026). Highly virulent *Aerococcus viridans* activates the RIPK3 signaling pathway, inducing necroptosis and inflammation in bovine mammary epithelial cells and murine mammary tissue. Vet. Microbiol..

